# Creation of a highly stable direct electron transfer-type enzyme sensor by combining a hyperthermophilic dehydrogenase and natural electron mediator

**DOI:** 10.1007/s10529-025-03587-3

**Published:** 2025-04-21

**Authors:** Miku Maeno, Haruhiko Sakuraba, Toshihisa Ohshima, Shin-ichiro Suye, Takenori Satomura

**Affiliations:** 1 Advanced Interdisciplinary Science and Technology, Graduate School of Engineering, University of Fukui, 3-9-1 Bunkyo, Fukui, 910-8507 Japan; 2 Department of Applied Biological Science, Faculty of Agriculture, Kagawa University, Kagawa, 761-0795 Japan; 3 Department of Biomedical Engineering, Osaka Institute of Technology, Osaka, 535-8585 Japan; 4 Division of Engineering, Faculty of Engineering, University of Fukui, 3-9-1 Bunkyo, Fukui, 910-8507 Japan; 5 Life Science Innovation Center, University of Fukui, 3-9-1 Bunkyo, Fukui, 910-8507 Japan

**Keywords:** Biosensor, Direct electron transfer, Fusion protein, Mediated electron transfer

## Abstract

This study aimed to address the stability limitations of third-generation biosensors using enzymes from mesophilic organisms, by engineering a stable direct electron transfer (DET)-type dehydrogenase capable of transferring electrons extracted from the substrate to the electrode. A fusion protein combining the mediated electron transfer (MET)-type aldose sugar dehydrogenase from the hyperthermophile *Pyrobaculum aerophilum* (PaeASD), which cannot transfer electrons generated by enzymatic reactions to the electrode without a mediator, and the natural electron transfer protein cytochrome *b*_562_ (cyt *b*_562_) was developed to investigate its potential for the DET reaction. A recombinant protein expression system was established in *Escherichia coli* to produce the PaeASD-cyt *b*_562_ fusion protein, which was purified from the soluble fraction of the host cells. Intramolecular electron transfer from pyrroloquinoline quinone (PQQ) to the heme group within the PaeASD-cyt *b*_562_ fusion protein was investigated using UV–Vis absorption spectroscopy. Upon the addition of glucose, an increase in absorption corresponding to reduced heme molecules was observed, indicating electron transfer from glucose to the heme group in the cyt *b*_562_ component via PQQ in the PaeASD component. The DET capability of the fusion protein was evaluated using cyclic voltammetry with screen-printed carbon electrodes. A glucose concentration-dependent increase in current response confirmed DET activity. Notably, the fusion protein retained over 80% of its initial current response even after 2 months of storage at 4 °C. The novel robust PaeASD-cyt *b*_562_ fusion protein demonstrated efficient DET capability, highlighting its high potential for application in the development of third-generation biosensors.

**Supplementary Information:**

The online version contains supplementary material available at 10.1007/s10529-025-03587-3.

## Introduction

Recent developments in electrochemical enzyme sensors have led to more selective and sensitive measurements of sample components in various fields, such as clinical diagnosis, food analysis, and environmental assessment (Vigneshvar et al. 2016; Sumitha and Xavier 2023).

Electrochemical enzyme sensors have attracted considerable attention as useful devices for this purpose. In these sensors, the substrate decrease accompanying the enzymatic reaction is detected as an electrical signal on an electrode. Oxidoreductases immobilized on the electrode extract electrons from the substrate through an oxidation reaction, and these extracted electrons are transferred to the electrode. The electrode can then detect the received electrons as current values corresponding to the substrate concentration. Such electrochemical enzyme sensors have been developed across the first to third generations (Das et al. 2016). In the first-generation biosensors, the amount of oxygen consumed or hydrogen peroxide produced by the oxidase is detected electrochemically. In this case, electrochemical detection errors are sometimes observed because of differences in the oxygen concentration in the sample solution.

In the second generation biosensors, a mediated electron transfer (MET) reaction is used to facilitate electron transfer between the enzyme and electrode. Different types of redox mediators such as potassium ferricyanide and phenazine methosulfate can be used for the MET reaction from the substrate to the electrode associated with the enzyme reaction. Mediators facilitate electron transfer from the substrate to the electrode via oxidoreductases; however, the tendency of redox mediators to leach into the sample solution and diffusion barriers at the enzyme-electrode interface may compromise the stability and reproducibility of second-generation biosensors. The development of third-generation biosensors is currently the focus of research. Third-generation biosensors use direct electron transfer (DET) enzymes as electrode elements and do not require oxygen molecules or redox mediators. These sensors are expected to overcome the limitations inherent in the first- and second-generation biosensors. Consequently, third-generation biosensors are expected to develop into new types of sensors. A previously reported DET-type sensor utilizes an enzyme with DET capability as an electrode element by modifying MET-type dehydrogenase with an artificial redox mediator (Hatada et al. 2018; Hiraka et al. 2020; Ikegai et al. 2024). Another example is the use of DET enzymes, which are genetically engineered fusions of cytochrome, a natural redox mediator, with MET-type enzymes (Algov et al. 2017; Yanase et al. 2020). However, the oxidoreductases useful for DET remain limited. The MET-type enzymes used in DET systems reported to date have all been obtained from mesophilic bacteria, demonstrate low stability, and are unsuitable for long-term use or storage. Recently, we succeeded in preparing a mutant of a highly thermostable PQQ-dependent aldose sugar dehydrogenase (mPaeASD) from the hyperthermophilic archaeon *Pyrobaculum aerophilum* (Maeno et al. 2025). In this study, we developed a novel robust DET-type enzyme from the highly stable MET-type mPaeASD in third-generation biosensors.

## Materials and methods

### Materials

Pyrroloquinoline quinone disodium salt (PQQ) was purchased from Fujifilm Wako (Osaka, Japan). Tryptone and yeast extract were purchased from Nacalai Tesque (Kyoto, Japan). Screen-printed carbon electrodes (SPCE, DS-110) were purchased from Metrohm-DropSens (Oviedo, Spain) and consisted of carbon as the working and counter electrodes and silver as the reference electrode. The working electrode had a diameter of 4 mm (surface area: 0.126 cm^2^). All the other chemicals were of reagent grade.

### Construction of the expression vector

To express the cytochrome *b*_562_ (cyt *b*_562_) fusion enzyme combined with PQQ-dependent aldose dehydrogenase (PQQ-ASD) at the C-terminus, a pET11a-mPaeASD-cyt plasmid was constructed using the pET-11a vector backbone (Novagen, Madison, WI, USA). mPaeASD is a double mutant of PQQ-ASD, containing the R64Q and D350N amino acid substitutions, which exhibit a six-fold increase in enzyme activity compared to wild-type PQQ-ASD (Maeno et al. 2025). A 1.6-kilobase gene fragment encoding the pelB signal sequence for periplasmic expression, the mPaeASD, an SGGGGSGGGGSGGGGS encoding linker sequence (Chen et al. 2013), cyt *b*_562_ without the signal sequence from *E. coli* B (residues 23–128) (Nikkila et al. 1991), and His-tag sequence were synthesized by TWIST Bioscience (San Francisco, CA, USA). The gene fragment containing the PQQ-ASD and cyt *b*_562_ fusion protein-encoding genes was amplified from the synthetic gene fragment by PCR, using the following primer pair: 5′- GAAGGAGATATACATATGAAATACCTG-3′ (forward) and 5′- TTAGCAGCCGGATCCTCAGTGGTGGTGGTGGTG-3′ (reverse). The forward and reverse primers contained the pET-11a sequence. pET-11a was linearized by inverse PCR using the following primer pair: 5′- GGATCCGGCTGCTAACAAAGCCCGAAAGGA-3′ (forward) and 5′-ATGTATATCTCCTTCTTAAAGTTAAACAAA-3′ (reverse) using PrimeSTAR Max DNA polymerase (Takara Bio, Shiga, Japan). After digestion of the intact pET-11a template with *Dpn* I, the amplified target gene fragments were inserted into the linearized pET-11a using an In-Fusion HD cloning kit (Takara Bio, Shiga, Japan) according to the manufacturer’s protocol. The nucleotide sequence reported in this paper has been submitted to the DDBJ databank under accession number LC853066.

### Expression and purification of the recombinant protein

To express the recombinant protein, *E. coli* BL21-CodonPlus (DE3)-RIPL (Agilent Technologies, Santa Clara, CA, USA) was transformed with pET11a-mPaeASD-cyt plasmid. The transformants were cultivated at 37 °C in 5 mL LB medium containing 50 μg mL^−1^ ampicillin. The pre-cultured transformants were inoculated in LB medium (1 L) containing ZYP-5052 medium (0.5% (w/v) glycerol, 0.05% (w/v) glucose, 0.2% (w/v) lactose, 50 mM KH_2_PO_4_, 25 mM (NH_4_)_2_SO_4_, 50 mM Na_2_HPO_4_, and 1 mM MgSO_4_ and 50 μg mL^−1^ ampicillin (Studier 2005; Sumikama et al. 2022). The transformants were incubated for 21 h at 30 °C with shaking. The cells were then harvested by centrifugation and washed twice with 0.85% NaCl solution. The washed cells were stored at −20 °C until further use.

The stored cells were suspended in 10 mM Tris–HCl (pH 8.0) buffer containing 20 mM imidazole and 100 mM NaCl and disrupted by ultrasonication to purify the recombinant protein. The lysate was centrifuged (10,000×g, 10 min) and the supernatant was applied to a 5 mL HisTrap FF Crude column (Cytiva, Tokyo, Japan) equilibrated with 10 mM Tris–HCl buffer (pH 8.0) containing 20 mM imidazole and 100 mM NaCl. The column was washed with three volumes of the same buffer. The enzyme was eluted using a linear gradient of 20–500 mM imidazole in the same buffer, over a three-bed volume. The apoenzyme was reconstituted as described previously (Sakuraba et al. 2010). Oxidation of the partially reduced cyt *b*_562_ expressed in the periplasmic fraction of *E. coli* was performed as previously described (Yanase et al. 2020).

### Enzyme activity and absorption spectrum analysis

The PQQ-ASD activity of the fusion protein was determined using a UV–VIS spectrophotometer (UV1800, Shimadzu, Japan) as previously described (Sakuraba et al. 2010). The enzyme assay was performed using 50 mM Tris–HCl (pH 8.0) containing 0.1 mM 2,6-dichloroindophenol (DCIP) and 500 mM glucose at 50 °C. The enzyme activity was determined by monitoring the decrease in absorbance at 600 nm accompanying DCIP reduction based on the molar absorption coefficient of DCIP (ε_mM_ 21.5 mM^−1^ cm^−1^ at 600 nm).

The absorption spectrum of the cyt *b*_562_ fusion protein was measured using a UV–VIS spectrophotometer. The fusion protein solution was prepared in 10 mM Tris–HCl buffer (pH 8.0) and reduced by adding 500 mM glucose.

### Electrochemical analysis

Cyclic voltammetry (CV) was performed using an electrochemical analyzer (Model 1205 B, BAS, Inc., Tokyo, Japan) and SPCEs. The electrolyte solution (25 μL) containing 50 mM Tris–HCl (pH 8.0), 0.5 mM enzyme, and d-glucose (1–250 mM) was dropped onto the top of the working carbon electrode. CV was conducted in the potential range of 0 to + 0.6 V at a scan rate of 1 mV s^−1^ at 30 °C.

## Results and discussion

### Design of DET-type mPaeASD-cyt *b*_562_ and expression of the fusion protein in *E. coli*

**Fig. 1:**
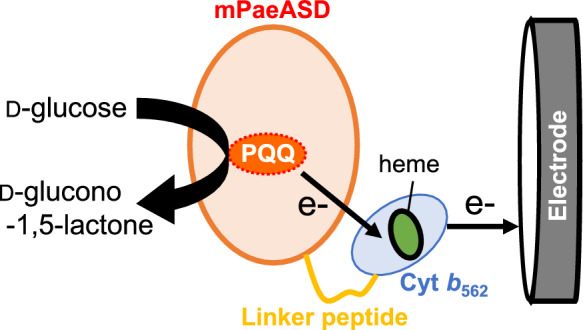
Schematic illustration of mPaeASD-cyt *b*_562_ and its electron transfer pathway

To create a DET-type dehydrogenase, we constructed the mPaeASD-cyt *b*_562_ recombinant protein expression system in which the natural electron acceptor cyt *b*_562_ (14 kDa) from *E. coli* was fused with the C-terminus of mPaeASD (38 kDa) (Fig. 1).

To prepare the fusion protein, mPaeASD was linked with cyt *b*_562_ via a linker peptide of 16 amino acid residues: SGGGGGSGGGGGSGGGGS. This peptide exhibited high flexibility and enabled efficient electron transfer between proteins by allowing unhindered interactions between mPaeASD and cyt *b*_562_. Furthermore, as the heme in the cyt *b*_562_ protein matures in the periplasmic fraction of *E. coli* (Nikkila et al. 1991), and the pelB signal sequence, a signal sequence for expression, promotes transport of the recombinant protein into the *E. coli* periplasmic fraction, the peptide-coding sequence was placed upstream of the gene encoding the fusion protein.

The expression vector carrying pET11a-mPaeASD-cyt was transformed into *E. coli* cells to produce the mPaeASD-cyt *b*_562_ fusion protein. The resulting *E. coli* carrying the recombinant mPaeASD-cyt *b*_562_ fusion protein expression vector exhibited a typical heme-derived reddish color.

A reddish color was also observed in the purified enzyme solution after nickel column chromatography. SDS-PAGE showed that the molecular weight of the purified protein after nickel affinity chromatography was consistent with that of mPaeASD-cyt *b*_562_ fusion protein (55 kDa) (Supplementary Fig. 1). The red coloration is likely attributable to the heme within cyt *b*_562_, which is expressed and matured in the periplasmic fraction of *E. coli*, indicating that cyt *b*_562_ is correctly expressed in *E. coli* cells.

### Redox activity of mPaeASD-cyt *b*_562_ fusion protein

The PQQ-ASD activity of the purified mPaeASD-cyt *b*_562_ fusion protein was measured spectrophotometrically using DCIP as the electron acceptor and showed a specific activity of 1.33 units mg^−1^. However, the specific activity of mPaeASD without cyt *b*_562_ was 11 units mg⁻^1^ (Maeno et al. 2025), which was approximately eight times higher than that of the mPaeASD-cyt *b*_562_ fusion protein. This suggests that, in the enzyme activity assay using the fusion protein with DCIP as an indicator, cyt *b*_562_ and DCIP competitively accepted the electrons produced by the d-glucose oxidation in the mPaeASD reaction, resulting in a decrease in apparent enzyme activity.

**Fig. 2:**
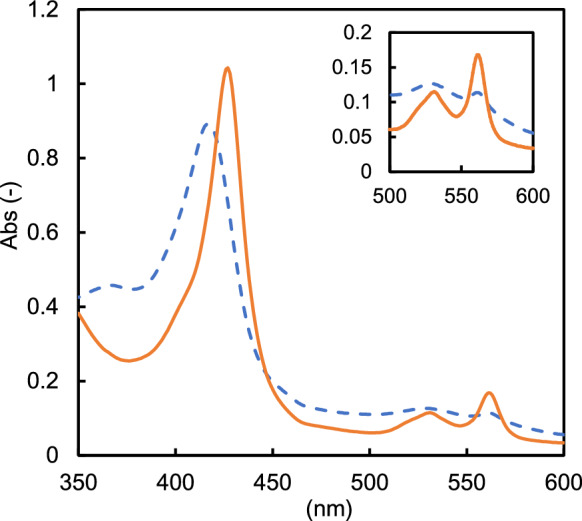
Spectroscopic observation of mPaeASD-cyt *b*_562_. mPaeASD-cyt *b*_562_ was subjected to spectroscopic analyses. Broken lines show the spectrum of the sample in its oxidative status after potassium ferricyanide treatment, and solid lines show the spectrum after glucose addition; an increase in the Q-band was observed in mPaeASD-cyt *b*_562_

The intramolecular electron transfer between PQQ-ASD and fused cyt *b*_562_ was then examined spectroscopically. The addition of glucose (500 mM) to the oxidized form of the mPaeASD-cyt *b*_562_ fusion protein resulted in an increase absorbance with two peaks at 540 and 560 nm, corresponding to the Q band derived from the reduced heme molecule (Fig. 2). This shows that the electrons generated by glucose oxidation with mPaeASD are transferred via PQQ in the mPaeASD and cyt *b*_562_ components of the fused protein, and finally to the heme molecule.

### DET ability of mPaeASD-cyt *b*_562_

**Fig. 3:**
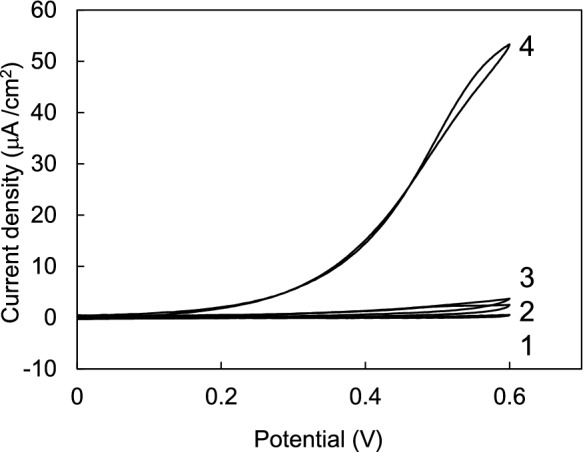
Comparison of cyclic voltammograms for mPaeASD and mPaeASD-cyt *b*_562_ using a screen-printed electrode. (1) mPaeASD without glucose, (2) mPaeASD with 250 mM glucose, (3) mPaeASD-cyt *b*_562_ without glucose, and (4) mPaeASD-cyt *b*_562_ with 250 mM glucose

**Fig. 4:**
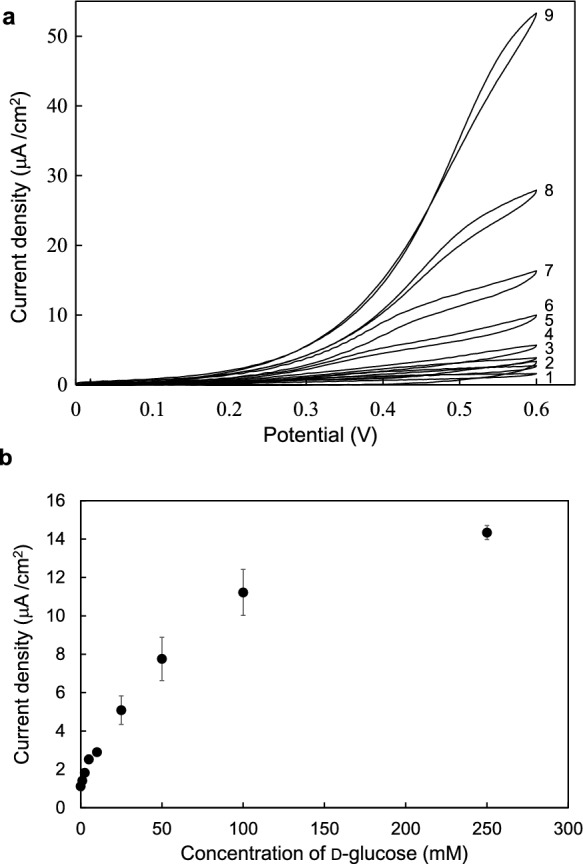
Cyclic voltammograms of mPaeASD-cyt *b*_562_ using screen-printed carbon electrodes. **a** Cyclic voltammograms of mPaeASD-cyt *b*_562_ in the presence of glucose: (1) 0 mM (2) 1 mM, (3) 2.5 mM, (4) 5 mM, (5) 10 mM, (6) 25 mM, (7) 50 mM, (8) 100 mM, and (9) 250 mM glucose. **b** Voltametric current responses of mPaeASD-cyt *b*_562_ in the presence of different glucose concentrations at + 0.4 V. Error bars indicate standard deviations (n = 3)

The DET ability of mPaeASD-cyt *b*_562_ cells was investigated by cyclic voltammograms (CVs) using the SPCEs. As illustrated in Fig. 3, CV revealed that the current response to glucose was barely detectable in the presence of mPaeASD alone. In contrast, a significant increase in the current response was observed with glucose when the mPaeASD-cyt *b*_562_ fusion protein was used. These findings unequivocally demonstrate that DET occurs exclusively upon fusion of cyt *b*_562_ with mPaeASD, underscoring the essential role of cyt *b*_562_ in mediating DET between the enzyme and electrode. In addition, Fig. 4a shows the CVs of mPaeASD-cyt *b*_562_ in solutions containing different concentrations of glucose (1–250 mM). As shown in Fig. 4b, the current densities at + 0.4 V increased in a glucose concentration-dependent manner. The apparent *K*_m_ and maximal current (*I*_max_) for d-glucose with mPaeASD-cyt *b*_562_ as an electrode element were determined to be 53.8 mM and 17.1 μA/cm^2^, respectively.

In general, MET-type dehydrogenases, such as mPaeASD, require an artificial electron mediator, such as DCIP, potassium ferricyanide or phenazine methosulfate, for electron transfer from the enzyme to the electrode (Maeno et al. 2025). However, the CV of the mPaeASD-cyt *b*_562_ fusion protein showed a glucose concentration-dependent increase in current without any artificial electron mediator. This indicates that mPaeASD-cyt *b*_562_ exhibits efficient DET ability.

When the storage stability of the mPaeASD-cyt *b*_562_ fusion protein was evaluated using CV with SPCE, approximately 80% of the initial current was retained after storage at 4 °C in 10 mM Tris–HCl buffer (pH 8.0) for 2 months.

Long-term use of an enzyme sensor may depend on the stability of the proteins used in the sensor system. Although MET-type dehydrogenases via cyt *b*_562_, which carry out the electron-transfer reaction between the enzyme and the electrode, have been reported previously, their dehydrogenase components originated from mesophiles (Okuda et al. 2003; Yanase et al. 2020). Among them, glucose dehydrogenase from *Acinetobacter calcoaceticus* loses activity after 10 min at 45 °C and lacks sufficient stability for practical use (Yoshida et al. 1999). In contrast, mPaeASD used in this study remained active even after 10 min at 95 °C (Maeno et al. 2025). Similarly, *E. coli* cyt *b*_562_ exhibits relatively high stability, with a half-life of 30 min at 67.2 °C (Feng and Sligar 1991). Therefore, the utilization of highly stable MET-type dehydrogenases could facilitate the development of more stable DET-type enzymes. In this study, we successfully created a highly stable DET-type enzyme by fusing cyt *b*_562_ with hyperthermostable mPaeASD, and showed that the fused enzyme may be a more useful element for electrochemical devices. Several highly stable MET-type dehydrogenases have been previously identified. Therefore, our results may promote further use of other thermostable MET-type dehydrogenases with cyt *b*_562_ for developing novel electrochemical devices.

## Conclusion

To develop a novel DET-type dehydrogenase with high stability, we established a recombinant expression system in *E. coli* for a fusion protein combining the hyperthermophilic MET-type dehydrogenase mPaeASD, and the natural electron transfer protein cyt *b*_562_. Upon adding glucose to the expressed recombinant protein mPaeASD-cyt *b*_562_, absorption increased because of reduced heme, confirming the intramolecular electron transfer from glucose to heme in the cyt *b*_562_ component via PQQ in the mPaeASD component of the fusion protein. The DET ability of mPaeASD-cyt *b*_562_ was assessed by CVs using SPCE, which revealed a glucose concentration-dependent increase in the current response, indicating the efficient DET capabilities of mPaeASD-cyt *b*_562_. Further, the current response of mPaeASD-cyt *b*_562_ on SPCE after 2 months storage at 4 °C was retained at more than 80% of the initial current response. The novel robust DET dehydrogenase indicates high potential for application in the development of third-generation biosensors with a long lifespan.

## Supplementary Information

Below is the link to the electronic supplementary material.

Supplementary file1 (DOCX 98 kb)

## Data Availability

Data will be made available on request.
