# Supplementary figures and images for: Creation of a highly stable direct electron transfer-type enzyme sensor by combining a hyperthermophilic dehydrogenase and natural electron mediator
Source: Biotechnol Lett. 2025 Apr 21;47(3):45. doi: 10.1007/s10529-025-03587-3 (PMC12011955; doi:10.1007/s10529-025-03587-3)

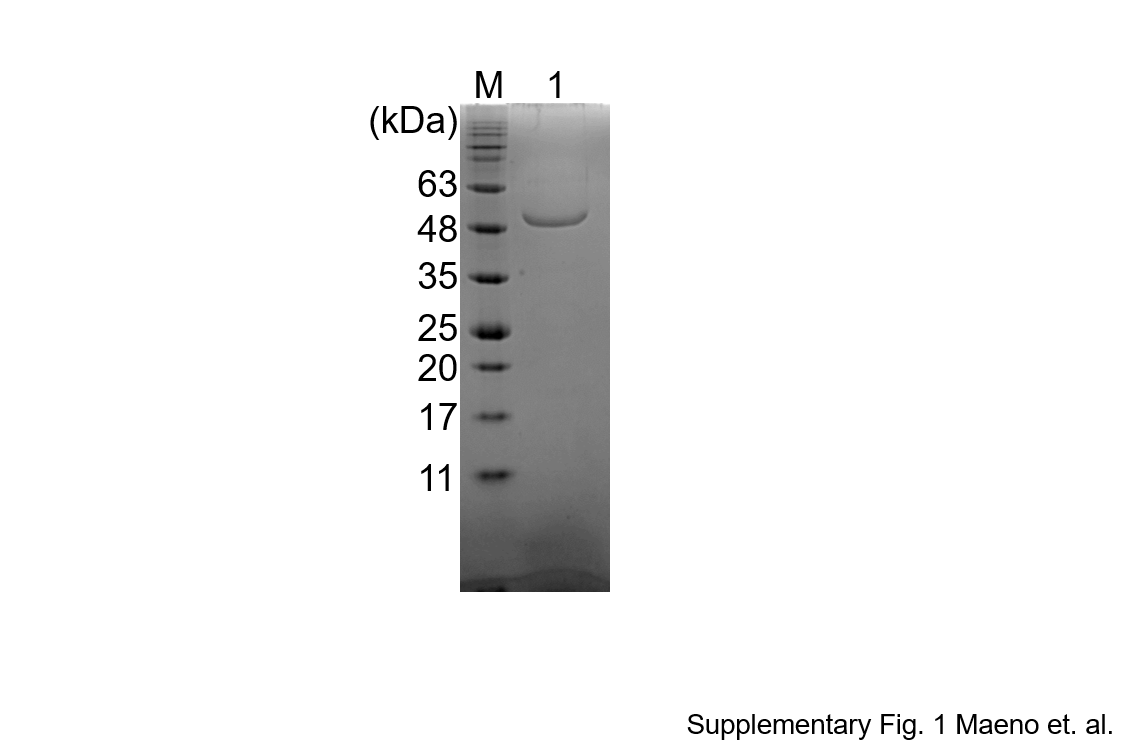

Supplement: Supplementary file 1 — Supplementary file1 (DOCX 98 kb) [file 10529_2025_3587_MOESM1_ESM.docx]
